# Editorial: Peptide Signaling in Plants

**DOI:** 10.3389/fpls.2022.843918

**Published:** 2022-02-15

**Authors:** Qingyu Wu, Wolfgang Schmidt, Reidunn Birgitta Aalen, Cao Xu, Fuminori Takahashi

**Affiliations:** ^1^Institute of Agricultural Resources and Regional Planning, Chinese Academy of Agricultural Sciences, Beijing, China; ^2^Institute of Plant and Microbial Biology, Academia Sinica, Taipei, Taiwan; ^3^Department of Biosciences, University of Oslo, Oslo, Norway; ^4^State Key Laboratory of Plant Genomics, CAS-JIC Centre of Excellence for Plant and Microbial Science (CEPAMS), Institute of Genetics and Developmental Biology, Chinese Academy of Sciences, Beijing, China; ^5^Faculty of Advanced Engineering, Tokyo University of Science, Tokyo, Japan

**Keywords:** peptide, plant development, CLE, TDIF, abiotic stress

Small peptides, molecules that consist of <100 amino acids, control a plethora of important biological processes in plants, including development, nutrient signaling, and stress responses. Although significant progress has been made over the past two decades in deciphering plant peptide signaling pathways, many facets of the biogenesis, function, and molecular mechanisms by which peptides act in plants remain to be clarified.

The articles in this Research Topic encompass an evolutionary comparison of different peptide families from a wide range of species, large-scale identification of novel peptides using a combination of multi-omics techniques, an analysis of peptide and receptor interactions using biochemical and molecular genetic approaches, and reviews summarizing recent findings in the field of plant peptides. Through the efforts of the contributors to this Research Topic, we have achieved a wider and deeper understanding of peptides in plants.

Despite significant progress, identifying small peptides remains challenging. Although bioinformatics approaches already in 2006 postulated the presence hundreds of Arabidopsis genes encoding small secreted peptides (Lease and Walker, 2006), relatively small number of peptide genes have been identified and functionally characterized. Thus, the number of peptides available for use in training sets for identifying new peptides is limited. As reviewed by Ren et al. many unconventional small peptides are derived from undocumented regions of the genome, such as intergenic regions, untranslated regions, introns, reading frames differing from those of annotated genes, and non-coding RNAs, rather than from well-annotated coding sequences, further hampering the identification of small peptides. New approaches are therefore needed to identify peptides at scale. Liang et al. identified 2,695 peptides by integrating a large-scale dataset that includes mRNA-seq, Ribo-seq, and mass spectrometry data from six tissues of maize (*Zea mays*). This study expands the inventory of known plant peptides. Unfortunately, many small peptides seem to function at extremely low concentrations in plants, and are difficult to detect due to technical limitations. Thus, a key goal in the field is developing highly sensitive technologies capable of cataloging these low-abundance peptides.

Examples of peptides that perform critical biological functions but are maintained at very low concentrations are members of the CLAVATA3/EMBRYO SURROUNDING REGION-RELATED (CLE) family. CLAVATA3 (CLV3), a peptide consisting of 12 or 13 amino acids in its mature form, regulates shoot meristem maintenance in the classical CLAVATA–WUSCHEL pathway (Clark et al., 1995) and was the first characterized member of this family. This pathway includes a secreted peptide, CLV3; a leucine-rich repeat receptor-like kinase, CLV1; and a homeodomain transcription factor, WUSCHEL (WUS). Binding of the CLV3 peptide by CLV1 leads to repression of WUS, which in turn promotes *CLV3* expression, thereby forming a negative feedback pathway [reviewed by (Li et al., 2021)]. This pathway is functionally conserved among a subset of flowering plants, including *Arabidopsis*, rice (*Oryza sativa*), tomato (*Solanum lycopersicum*), maize, and *Brassica juncea*. Works by Zhu et al. and Takahashi et al. expand our knowledge of the CLV–WUS pathway to a larger group of species. Zhu et al. show that ablation of *Setaria viridis* FLORAL ORGAN NUMBER 2 (SvFON2), the homolog of *Arabidopsis* CLV3 and rice FON2 peptides, results in a larger inflorescence meristem, suggesting that SvFON2 functions in inflorescence meristem development. Results reported by Takahashi et al. revealed that, similar to the *Arabidopsis* model, the receptor MpCLV1 and co-receptor MpCLAVATA3 INSENSITIVE RECEPTOR KINASE (MpCIK) form a complex that perceives signals from the *Marchantia polymorpha* CLV3 ortholog MpCLE2, suggesting that the function of CLV3-like peptides and their receptors is conserved beyond flowering plants.

Members of the CLE family, such as DODECAPETIDE TRACHEARY ELEMENT DIFFERENTIATION INHIBITORY FACTOR (TDIF)/CLE41, are also important regulators of vascular development, as reviewed by Yuan and Wang. TDIF/CLE41 binds to its receptor PHLOEM INTERCALATED WITH XYLEM/TDIF RECEPTOR (PXY/TDR) and signals through two WUSCHEL-related HOMEOBOX transcription factors, WOX4 and WOX14 (Hirakawa et al., 2010; Etchells et al., 2013). Xu et al. identified a new regulator in the CLE41/TDIF-TDR/PXY signaling pathway: PXY-CORRELATED 3 (PXC3). The width of the hypocotyl stele and the number of vascular cells are reduced in *pxc3* loss-of-function mutants, indicating that PXC3 functions in vascular development in *Arabidopsis*. Tian et al. demonstrate that ectopic expression of the switchgrass *CLE* family member *DODECAPETIDE TRACHEARY ELEMENT DIFFERENTIATION INHIBITORY FACTOR-LIKE 1* (*PvTDIFL1*) in *Arabidopsis* strongly inhibits plant growth, increases cell division in vascular tissue, and disrupts the cellular organization of the hypocotyl, suggesting that TDIFL proteins are functionally conserved between switchgrass and *Arabidopsis*.

Our knowledge of the evolutionary processes that gave rise to small peptides is limited. To address this shortcoming, Furumizu and Sawa searched for members of the ROOT GROWTH FACTOR (RGF)/GOLVEN (GLV)/CLE-LIKE (CLEL) family of peptides in a wide range of species, including liverworts, mosses, hornworts, lycophytes, and ferns. All major extant land plant lineages, except hornworts, harbored subsets from a total of more than 400 identified RGF-like peptides. The authors demonstrate that MpRGF from *Marchantia polymorpha* possesses known RGF-like activities and can affect plant growth when constitutively expressed in *Arabidopsis* or *Marchantia polymorpha*. This study advances our understanding of how peptide signaling pathways evolved.

In addition to their developmental functions, plant peptides are involved in abiotic stress responses via cell-to-cell communication networks (Kim et al.). In their review, the authors summarize the roles of different peptide families in orchestrating plant responses to drought, salt, heat, nutrient deficiency, and reactive oxygen species (ROS). Remarkably, the ability of plants to modulate ROS production allows small peptides to regulate pollen tube growth. Kou et al. show that adding purified phytosulfokine PSK2 to pollen promotes pear pollen tube elongation in a dose-dependent manner by increasing ROS production, indicating a potential application of peptides in improving pollination efficiency.

Collectively, this Research Topic provides fascinating and topical information on plant peptide signaling. The articles cover important aspects of plant peptides, such as their involvement in plant development and abiotic stress, and the mining of peptides from multi-omics data. However, several questions remain to be addressed. How can low-abundance peptides be detected efficiently? How do receptors recognize and distinguish different peptides that share high sequence similarity? Can external application of small peptides improve crop yield and tolerance to biotic and abiotic stresses? Can small peptides be tweaked to improve agronomic traits and increase agricultural sustainability? Given the potential benefits that answers to these questions may hold, we would like to invite more scientists to join us in studying these impressive and intriguing small molecules.

## Author Contributions

All authors wrote and approved the editorial.

## Conflict of Interest

The authors declare that the research was conducted in the absence of any commercial or financial relationships that could be construed as a potential conflict of interest.

## Publisher's Note

All claims expressed in this article are solely those of the authors and do not necessarily represent those of their affiliated organizations, or those of the publisher, the editors and the reviewers. Any product that may be evaluated in this article, or claim that may be made by its manufacturer, is not guaranteed or endorsed by the publisher.
